# Palmitoylethanolamide (PEA) Induces an Increase in Spleen Regulatory T Cells, Reduces CD8 ^+^ Cells and TNF‐α Levels in Target Organs, and Protects Mice From Graft‐Versus‐Host Disease‐Related Mortality Through PPAR Activation Without Compromising the Graft‐Versus‐Tumour Response

**DOI:** 10.1111/imm.70010

**Published:** 2025-06-25

**Authors:** Bárbara Betônico Berg, Zara Desiree Tonidandel Campos, Gioconda Muniz Fiorenza Ruggio, Ana Flávia Santos Linhares, Bárbara Maximino Rezende, Stêfany Bruno de Assis Cau, Thiago Roberto Lima Romero, Mauro Martins Teixeira, Vanessa Pinho, Marina Gomes Miranda Castor

**Affiliations:** ^1^ Graduate Program in Biological Sciences: Physiology and Pharmacology Federal University of Minas Gerais Belo Horizonte Brazil; ^2^ Department of Nursing, School of Nursing Federal University of Minas Gerais Belo Horizonte Brazil; ^3^ Department of Pharmacology Federal University of Minas Gerais Belo Horizonte Brazil; ^4^ Department of Biochemistry and Immunology Federal University of Minas Gerais Belo Horizonte Brazil; ^5^ Department of Morphology, Institute of Biological Sciences Federal University of Minas Gerais Belo Horizonte Brazil

**Keywords:** endocannabinoids, graft‐versus‐host disease, inflammation

## Abstract

Graft‐versus‐host disease (GVHD), a secondary complication of bone marrow transplantation, leads to the development of a systemic inflammatory illness in the host, resulting in high mortality and morbidity. Current therapies lack prophylactic effectiveness and often fail to achieve an optimal immunological balance between inflammation and immunosuppression. In this study, we investigated the effects of palmitoylethanolamide (PEA), an endocannabinoid‐like lipid mediator with extensively documented anti‐inflammatory, analgesic, antimicrobial, immunomodulatory, and neuroprotective effects, on the complex pathology of GVHD. Treatment with PEA reduced clinical disease severity in GVHD mice, leading to an 80% increase in survival rates. Additionally, PEA created an immunoregulatory environment in the spleen by reducing the activation of CD3^+^CD4^+^ cells. In the intestine, PEA protected against damage, reduced the number of CD3^+^CD4^+^ and CD3^+^CD8^+^ cells, and suppressed the activation of CD3^+^CD8^+^ cells. PEA also decreased the levels of TNF‐α in the intestine and increased IL‐10 production. Furthermore, in the liver, PEA treatment reduced the number of CD8^+^ cells, the activation of CD3^+^CD4^+^ and CD3^+^CD8^+^ cells, and TNF‐α levels. The effect of PEA on survival was dependent on Peroxisome Proliferator‐activated receptor gamma (PPAR‐γ) activation but did not rely on cannabinoid (CB) receptors activation. In addition to GVHD protection, PEA treatment did not interfere in the graft‐versus‐tumour response. These results demonstrate the therapeutic potential of PEA as a promising option for the treatment of GVHD, balancing inflammation and immunosuppression, and improving both survival and clinical outcomes.

## Graphical abstract

Effect of Palmitoylethanolamide (PEA) in a murine model of graft‐versus‐host disease (GVHD). Following an allogeneic transplant from C57BL/6J donors to BALB/c recipients, PEA treatment improved survival, alleviated clinical symptoms, and protected target organs in GVHD‐affected mice.

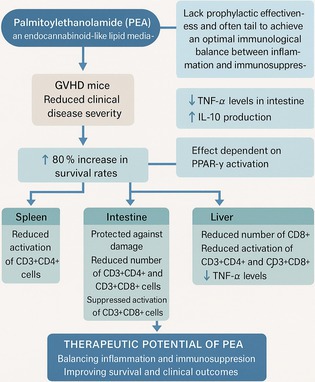

**Abbreviations**
Δ9‐THC: delta‐9‐tetrahidrocanabinol
AEA: arachidonoyl ethanolamine/anandamide
CB_1_
: cannabinoid receptor type 1
CB_2_
: cannabinoid receptor type 2
CBD–: cannabidiol
CCL: C‐C motif chemokine ligand
DMSO: dimethyl sulfoxide
ECS: endocannabinoid system
ELISA: enzyme‐linked immunosorbent assay
FACS: fluorescence‐activated cell sorting
FITC: fluorescein isothiocyanate
Fox: forkhead box
GFP: green fluorescence protein
GPCR: G protein‐coupled receptors
GVHD: graft‐versus‐host disease
GVT: graft‐versus‐tumour
Gγ: grey (unit of ionising radiation)
H&E: haematoxylin and eosin
H2‐Db: MHC Class I Allele B
H2‐Dd: MHC Class I Allele D
HSCs: haematopoietic stem cells
HSCT: haematopoietic stem cell transplantation
i.v.: intravenous
ICAM‐1: intercellular adhesion molecule 1
IFNγ: interferon γ
IL: interleukin
MFI: mean fluorescence intensity
MHC: major histocompatibility complex
NAEs: N‐acylethanolamines
PE: phycoerythrin
PEA: palmitoylethanolamide
PerCP: peridinin‐chlorophyll‐protein
TNFα: tumour necrosis factor α

## 1 Introduction

Haematopoietic stem cells (HSCs) are a class of transplantable tissue with significant regenerative capacity, primarily due to their ability to differentiate into various haematopoietic lineage components following appropriate stimulation. This capacity allows for the reconstitution of haematopoiesis by replacing the recipient's damaged cells with healthy donor cells [1]. Among the various transplantation approaches, allogeneic transplantation is particularly notable. The efficacy of allogeneic haematopoietic stem cell transplantation (HSCT) is largely attributed to the graft‐versus‐tumour (GVT) effect [2]. This occurs when transplanted allogeneic CD4^+^ T cells, through the secretion of interferon‐γ (IFN‐γ), enhance the proliferation and activation of cytotoxic CD8^+^ T cells, which then recognise and eliminate residual tumour cells in the host, thereby suppressing tumour growth [3]. Consequently, patients with hematologic malignancies benefit from eradicating residual cancer cells and preventing disease relapse [4].

However, the therapeutic success of allogeneic HSCT is often compromised by graft‐versus‐host disease (GVHD), a severe complication arising from genetic disparities between donor and recipient [5]. GVHD is characterised by a hyperinflammatory response, extensive tissue damage, and a cytokine storm, primarily involving the gastrointestinal tract, liver, skin, and lungs [6]. This condition is the leading cause of non‐relapse mortality post‐transplantation [7], and it contributes to clinicians' hesitancy to pursue this therapeutic approach [8].

The disease occurs due to the activation of T lymphocytes, which are triggered by antigen‐presenting cells (APCs) that recognise disparities between the donor and recipient cells, including both residual recipient cells and those derived from the transplanted marrow [9]. This cross‐presentation occurs via MHC‐I for CD8^+^ T cells and MHC‐II for cells such as CD4^+^ T lymphocytes [10]. Consequently, the expansion of cytotoxic effector alloreactive T cells, accompanied by a cytokine storm and the subsequent migration of these cells to target organs, perpetuates the inflammatory response and characterises the early stages of GVHD [11, 12].

Despite ongoing advances in medicine, the primary treatment for this disease remains prolonged use of immunosuppressants and corticosteroids [12, 13]. Approximately 60% of patients treated with corticosteroids become tolerant to the therapy and develop steroid‐refractory GVHD, which has a survival rate of 30% to 40% [14]. Immunosuppressants are also used prophylactically and during the conditioning regimen. This includes drugs such as alemtuzumab, an anti‐CD52 antibody [15, 16], and other agents like sirolimus [17], cyclosporine [18] or tacrolimus [19].

New therapies have been proposed complementary to corticosteroid use and potential replacements, such as CAR‐T cells or monoclonal antibodies like Inolimomab (CD25). CAR‐T cells are genetically modified cells that express chimeric antigens, enabling them to target specific receptors and cell types to reduce the inflammatory response [20]. The main drawback of this therapy, as well as that of monoclonal antibody therapy, is the high toxicity, with potential complications including cytokine release syndrome and neurological syndromes triggered by immune effector cells [21, 22]. Moreover, despite these effects, there remains a risk of GVHD, in which case corticosteroid use reverts to being the standard therapy [23].

In this context, therapies based on molecules with known immunomodulatory effects become interesting therapeutic targets. The pharmacological activities of PEA were first described in the mid‐1950s when Ganley and Robinson (1958) reported the initial evidence of anti‐inflammatory activity, identifying therapeutic properties in anaphylaxis. In 1971, the first report suggested that this exogenous compound could be helpful in the management of arthritis [24]. Currently, the literature provides evidence for its use as an immunomodulator [25], in analgesia [26], in the management of hypersensitivity [25], in alterations of central nervous system activities such as Alzheimer's disease [27], multiple sclerosis [28], and in sciatic nerve injuries [29]. Additionally, PEA shows significant potential as an anti‐inflammatory agent [30].

Studies have demonstrated that PEA exhibits activity on the CB_2_ receptor, playing an antinociceptive in vivo [31] and neuroprotective role in vitro [32], as well as on the colonic CB1 receptor as an anti‐inflammatory agent [33]. Furthermore, this lipid compound also promotes an increase in the concentration of AEA, thereby enhancing its action [34]. In addition, PEA acts by receptors such as PPARs [35, 36]. These therapeutic targets underscore the potential of PEA as an anti‐inflammatory and immunomodulatory tool [37].

In this context, the present study aims to investigate the therapeutic use of PEA in the inflammatory response associated with murine GVHD, as well as the correlation of its effects with its pharmacological targets.

## 2 Methods

### 2.1 Animals

BALB‐c mice, ranging from 8 to 12 weeks of age, were our research subjects. Both C57BL/6J and BALB‐c mice were used as bone marrow and splenocyte donors for the disease induction and control groups, respectively. The mice were provided by the Center of Bioterism of the Federal University of Minas Gerais, Belo Horizonte, Brazil, or the René Rachou Research Center of the Oswaldo Cruz Foundation, Belo Horizonte, Brazil. The animals were housed in controlled conditions under a 12 h light–dark cycle at a temperature of 25°C ± 2°C, with food and water available ad libitum. All procedures performed were approved by the Ethics Committee on Animal Experimentation of the Federal University of Minas Gerais under protocol number 203/2021. The experiments were carried out following the guidelines of the Guide for the Care and Use of Laboratory Animals and were in accordance with the Animal Research: Reporting of In Vivo Experiments (ARRIVE) guidelines.

### 2.2 Disease Induction

Using gamma radiation, BALB/c mice were subjected to two doses of 3.5 Gy for bone marrow ablation. Subsequently, these mice received 3 × 10^7^ splenocytes and 1 × 10^7^ bone marrow cells intravenously, either from an allogeneic C57BL/6J donor, thereby developing GVHD, or from syngeneic BALB/c mice, which did not develop the disease. All mice were administered ciprofloxacin for the first 7 days post‐transplant to prevent secondary infections [38, 39]. Following the transplant, the mice were monitored every 2 days using a standard clinical scoring system that evaluated and scored parameters such as hunching, activity, fur loss and texture, weight loss, skin integrity, and diarrhoea. The scoring scale varied according to clinical manifestations: 0 = normal; 0.5 = slight alteration; 1 = minor alteration; 1.5 = moderate alteration; and 2 = severe alteration. To minimise animal suffering, a standardised cut‐off level of 10 points was implemented [10, 38, 40, 41].

### 2.3 Pharmacological Treatments

Allogeneic transplanted mice received 1, 5 or 10 mg of PEA (Cayman Chemical Company, Michigan, USA) or its vehicle (1,25% of ethanol + 1,25% DMSO In PBS1X). The participation of CB1 and CB2 was assessed by treatment with 1 mg/kg of their respective Antagonist AM251 (Tocris, Sellex, Sao Paulo, SP, Brazil) or selective inverse agonists AM630 (Tocris, Sellex, Sao Paulo, SP, Brazil). Participation of PPAR‐γ receptor was verified by the PPAR‐γ antagonist GW 9662: 2‐chloro‐5‐nitrobenzanilide, dose of 1 mg/kg, dissolved in 2.5% DMSO in PBS1x. Brand: Tocris, Sellex, São Paulo, SP, Brazil. One hour before the treatment with target agonists, and both their vehicles consisted of 2.5% DMSO in PBS‐1x. All drugs were administered daily by intraperitoneal injection starting 2 h before bone marrow ablation and continuing for the duration of the protocols. The dose of AM251, AM630 and GW9662 were in accordance with previous studies [38, 42, 43].

### 2.4 Histopathological Analysis

Seven days after transplantation, the mice were euthanized and their livers and intestines were collected and fixed in formaldehyde before being paraffinised and stained with Hematoxylin and Eosin (H&E) (Abcam, Cambridge, MA, USA). Regions of the jejunum–ileum and the full extension of the liver were blindly analysed by a pathologist under an optical microscope (Olympus BX51, Tokyo, Japan) based on the following scoring system. In the jejunum–ileum, the scoring system assigned numerical values according to changes observed in the mucosa (0 = no alterations, 1 = reactive alterations, 2 = loss of architecture, 3 = nuclear changes and hyperplasia without ulceration or loss of goblet cells, 4 = nuclear changes and hyperplasia with ulceration and/or loss of goblet cells). Lamina propria as well as muscular and serosa layers were scored according to the presence of inflammatory infiltrate (0 = absent, 1 = discrete cellular infiltrate, 2 = moderate cellular infiltrate with or without swelling, 3 = severe inflammatory infiltrate with villous enlargement, swelling, and congestion in the lamina propria; 4 = ischemic necrosis and intense inflammatory infiltration mainly in muscular and serosa layers, 5 = ischemic necrosis and intense inflammatory infiltration with generalised loss of architecture). The sum of the individual results was used as the general score of the animal, with a maximum index of nine points.

For the liver, degenerative alterations in the parenchyma were enumerated as 0 = normal, 1 = discrete vacuolization of the cytoplasm and focal eosinophilia, 2 = diffuse vacuolization and alterations in hepatocyte and nucleus morphology, and 3 = necrosis and diffuse vacuolization, alterations in hepatocyte morphology, and the nucleus. Inflammatory infiltration was scored as: 0 = none, 1 = discrete infiltration in the periportal area, 2 = discrete or moderate presence of periportal and intralobular cellular infiltration, and 3 = accentuated cellular infiltration in periportal and intralobular regions. The sum of the values gave the mice's total score, which was a maximum index of six points.

### 2.5 Enzyme‐Linked Immunosorbent Assay (ELISA)

Mice were euthanized on the seventh day after transplantation. A 100 mg sample from the liver and intestine was collected and processed for ELISA assay. The concentrations of the cytokines CCL2, CCL3, CCL5, IL‐10, TNF‐α, and IFN‐γ were measured according to the manufacturer's instructions (R&D Systems, Minneapolis, MN, USA) at a wavelength of 490 nm using a spectrophotometer (Agilent BioTek ELx808 Absorbance Reader, Agilent Technologies, Barueri, SP, Brazil).

### 2.6 Flow Cytometry

Panels of gate strategy of Lymphocyte subtypes and their specific markers were provided in Figures S1 and S2. Tissue samples from the bone marrow (Figure S3), spleen (Figure S4), intestine (Figure S5), and liver (Figure S6) were collected and processed on Day 7 according to the protocol described by Berg et al. [38]. The number of cells was counted using a Neubauer chamber, and a total of 1 × 10^6^ cells were stained with the following antibodies: CD3 (Pacific Blue [RRID‐17‐A2], BioLegend, San Diego, USA), CD4 (PE‐Cy7 [RRID‐RM45], BioLegend), CD8 (FITC [RRID‐53‐6.7], BioLegend), CD25 (allophycocyanin [RRID‐PC61], BioLegend), and CD28 (PE‐A [RRID‐37.51], BioLegend), FoxP3 (PE‐A [RRID‐MF14], BioLegend), and CD62L (allophycocyanin [MEL‐14 Biolegend]). For the evaluation of the chimerism process, we used H2‐Db (AlexaFluor 647 [RRID‐KH95], BioLegend) and H2‐Dd (PE [RRID‐34,212], BioLegend) as markers in the bone marrow and spleen. A total of 1 million events were considered for each sample and tracked using a BD FACSCanto II cytometer (New Jersey, USA) and further analysed using FlowJo V10 software (BD Immunocytometry Systems, San Jose, USA). H2‐Db and H2‐Dd are MHC class I alloantigens expressed on all nucleated cells from mice that express either of these haplotypes, namely C57BL/6 and BALB‐c mice, respectively. These alloantigens are involved in CD3/TCR T cell antigen presentation.

### 2.7 GVT

GVHD was induced in the Balb/c mice as described in Section 2.2. At the GVHD induction, the mice received murine mastocytoma P815^+^ cells i.v. (H‐2d, American Type Culture Collection, Rockville, MD—RRID TIB‐64) transduced with the EF1aGFP vector (National Institute of Cancer, Rio de Janeiro, Brasil) in a concentration of 1 × 10^5^ cells. Mice were treated with vehicle or PEA for 7 days. The lymph nodes (mesenteric and inguinal) were processed for FACS analysis by Flow Cytometry (FacsScaibur). The number of tumour cells was evaluated by the percentage of GFP+ fluorescence.

### 2.8 Statistical Analysis

Data was analysed using GraphPad Prism 8.0.2 software. Normality was assessed by the Shapiro–Wilk test, and outliers were determined using Grubb's test (*α* = 0.05). Differences between vehicle and PEA treated groups were determined by the Unpaired parametric Two‐tailed Student's *t* test (*p* < 0.05). Differences in survival curves were determined based on the log‐rank (Mantel–Cox) test (*p* < 0.05). For the clinical score, two‐way ANOVA with Bonferroni's multiple comparison test was used for statistical hypothesis testing (*p* < 0.05).

## 3 Results

**FIGURE 1:**
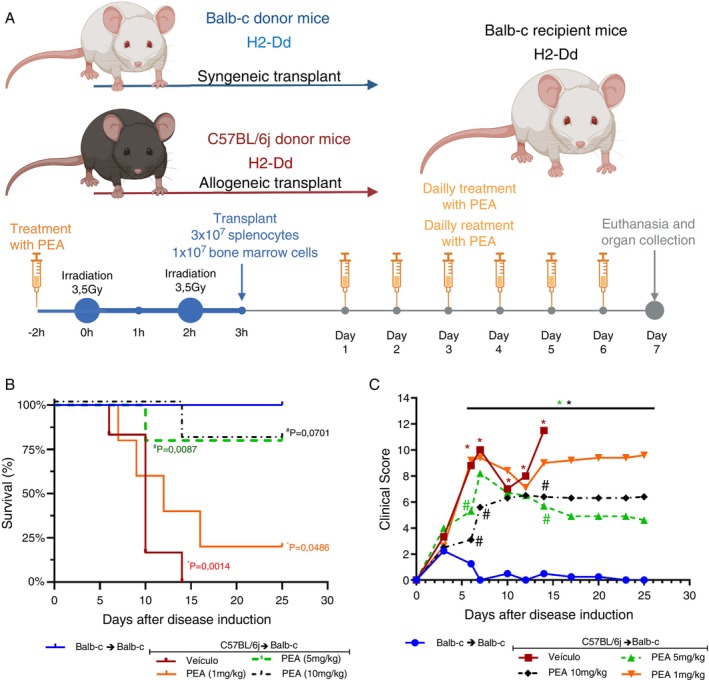
Evaluation of PEA treatment on survival and clinical parameters of acute murine Graft‐versus‐host disease. (A) Procedure of disease induction, previously irradiated BALB‐c mice received either allogeneic or syngeneic splenocytes (3 × 10^7^) and bone marrow cells (1 × 10^7^), thus developing or not GVHD, respectively. GVHD occurs due to differences in MHC I from BALB‐c (H2‐Dd) and C57BL/6J (H2‐Db) mice. (B) Percentage of survival of mice receiving daily treatment of PEA 1 mg/kg (orange line), PEA 5 mg/kg (green line), PEA 10 mg/kg (black line), compared to vehicle (red line) (#) or (*) compared to syngeneic transplanted (blue line) mice. (C) Clinical score of mice receiving daily treatment of PEA 1 mg/kg (orange line), PEA 5 mg/kg (green line), PEA 10 mg/kg (black line), compared to vehicle (red line) (#) compared to syngeneic transplanted (blue line) (*) mice. *n* = 10 for all groups and in all experiments. Treatment was performed from Day 0 until the end of the experiment. Survival was verified by Log‐rank (Mantel‐Cox) test and groups were considered different only when *p* value was < 0.05. Two‐way ANOVA with Bonferroni's multiple comparisons test was used to compare the clinical scores of groups, and they were considered different only when *p* value was < 0.05.

BALB‐c mice received the transplant from syngeneic (BALB‐c) or allogeneic (C57BL/6J) donor mice. As expected, the recipients of allogeneic transplants developed GVHD (Figure 1A), which was the focus of the study. The GVHD mice were treated with the target drugs from the day of disease induction until either the seventh day (for mice euthanized for analysis) or until mortality (for mice undergoing mortality evaluation). The mice were closely observed, and their clinical scores were recorded to assess their overall health and well‐being throughout the study. PEA 5 and 10 mg/kg increased survival at 80% compared to vehicle‐treated mice (Figure 1B) and reduced clinical signs of murine GVHD (Figure 1C).

### 3.1 PEA Treatment Did Not Change the Engraftment Process

**FIGURE 2:**
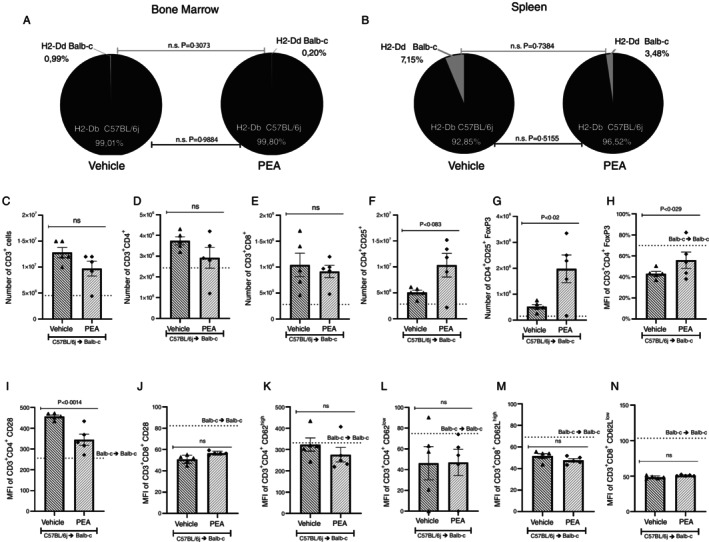
Evaluation of PEA treatment on engraftment process and chimerism in BALB‐c recipient mice. Schematic representation of the bone marrow and spleen engraftment process. The presence of mice MHC I H2‐Dd (BALB‐c) and H2‐Db (C57BL/6J) was verified by flow cytometry in bone marrow cells (A) and in spleen cells from Vehicle or PEA treated mice, 7 days after transplantation. The unpaired parametric two‐tailed Student's *t* test was used to assess differences between Vehicle and AEA, the groups were considered significantly different when *p* < 0.05 (*n* = 5 in all groups). Next, lymphocytes populations were analysed by flow cytometry, identifying (C) Number of CD3^+^ lymphocytes. (D) Number of CD3^+^CD4^+^ lymphocytes. (E) Number of CD3^+^CD8^+^ lymphocytes. (F) Number of CD4^+^CD25^+^ lymphocytes. (G) Number of CD4^+^CD25^+^ FoxP3^+^ lymphocytes. (H) Mean fluorescence intensity (MFI) of CD4^+^CD25^+^ FoxP3+. (I) MFI of CD28 in CD3^+^CD4^+^ lymphocytes. (J) MFI of CD28 in CD3^+^CD8^+^ lymphocytes. (K) MFI of CD3^+^CD4^+^ CD62L^high^ lymphocytes. (L) MFI of CD3^+^CD4^+^ CD62L^low^ lymphocytes. (M) MFI of CD3^+^CD8^+^ CD62L^high^ lymphocytes. (N) MFI of CD3^+^CD8^+^ CD62L^low^ lymphocytes. Treatment was performed from Day 0 until the end of the experiment. Groups were considered significantly different when *p* < 0.05, compared to allogeneic group treated with vehicle. Assessed by unpaired parametric two‐tailed Student's *t* test (*n* = 5 in all groups).

Bone marrow and spleen, 7 days after transplantation, were analysed using flow cytometry to verify if transplanted bone marrow cells had successfully transmigrated and colonised lymphoid organs. In both organs, we analysed the presence of BALB‐c MHC‐1 marker (H2‐Dd) and C57BL/6J MHC‐1 (H2‐Db). In allogeneic transplanted mice, it is crucial to find a predominance of H2‐Db. Treatment with PEA did not interfere with the engraftment process in bone marrow, and hence we found a predominance of H2‐Db (99.01% in the vehicle and 99.80% in PEA) over H2‐Dd (0.99% in the vehicle and 0.20% in PEA) (Figures 2A and S1). In the spleen, the same predominance of H2‐Db (92.85% in the vehicle and 96.52% in PEA) over H2‐Dd (7.15% in the vehicle and 3.48% in PEA) was found (Figures 2B and S2).

### 3.2 PEA Increased CD3 ^+^ CD4 ^+^ FOXP3 ^+^ Cells and Reduced Activation of CD3 ^+^ CD4 ^+^ Cells in the Spleen of GVHD Mice

The cellular populations in the spleen were evaluated to verify if the allogeneic transplant or treatment with PEA led to differences in cellular populations. The number of CD3^+^, CD3^+^CD4^+^, and CD3^+^CD8^+^ was increased in the vehicle when compared to the syngeneic group, but there was no difference between the vehicle and the PEA‐treated group (Figure 2C–E). Concerning the number of CD4^+^CD25^+^ cells and CD4^+^CD25^+^FoxP3^+^ cells, treatment with PEA increased these populations and increased the mean fluorescence intensity of FoxP3^+^ cells compared to vehicle treated mice (Figure 2F–H). Regarding lymphocyte activation status, marked by MFI of CD28, PEA treatment reduced the MFI of CD28 in the CD3^+^CD4^+^ cells compared to vehicle treated mice (Figure 2I). However, no difference was found between groups regarding CD3^+^CD8^+^CD28 cells (Figure 2J). The profile of effector or memory T cells was verified by a CD62L marker in the spleen. Treatment with PEA did not alter the CD3^+^CD4^+^CD62L^low^, effector memory T cells and CD3^+^CD4^+^CD62L^high^ central memory T cells, nor CD3^+^CD8^+^CD62L^low^ or CD3^+^CD8^+^CD62L^high^ populations in the spleen, compared to the vehicle group (Figure 2K–N).

### 3.3 PEA Reduced the Number of CD3 ^+^ CD4 ^+^ and CD3 ^+^ CD8 ^+^ Cells and the Activation of CD3^+^CD8^+^ in the Intestine, Reducing Damage, TNF‐α Levels and Increasing the Anti‐Inflammatory Cytokine IL‐10 in the Intestine of GVHD Mice

**FIGURE 3:**
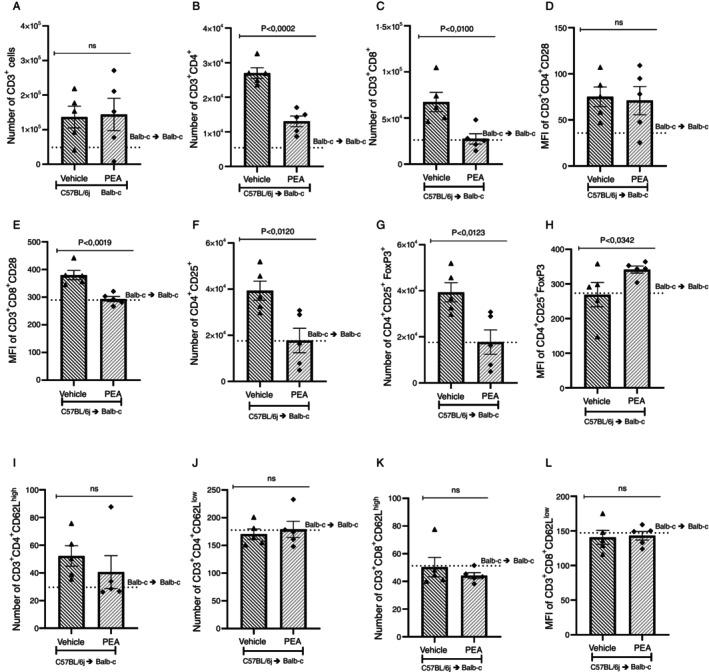
PEA reduces CD3^+^CD4^+^ and CD3^+^CD8^+^ cells infiltration and activation in the intestine of GVHD mice. The number and profile of infiltrated cells in the intestine were analysed through flow cytometry. (A) CD3^+^, (B) CD3^+^CD4^+^ and (C) CD3^+^CD8^+^ lymphocytes, (MFI) of CD28 was assessed in (D) CD3^+^CD4^+^ and (E) CD3^+^CD8^+^ cells. Furthermore, the number of (F) CD4^+^CD25^+^ cells and the (G) CD4^+^CD25^+^FoxP3^+^ cells and the (H) MFI of CD4^+^CD25^+^FoxP3^+^ were investigated. The profile of effector or central memory cells was also investigated in T cells (I) CD3^+^CD4^+^CD62L^high^, (J) CD3^+^CD4^+^CD62L^low^, (K) CD3^+^CD8^+^CD62L^high^ and (L) CD3^+^CD8^+^CD62L^low^. Treatment was performed from Day 0 until the end of the experiment. Groups were considered significantly different when *p* < 0.05, compared to the allogeneic group. Assessed by unpaired parametric two‐tailed Student's *t* test, (*n* = 5 in all groups).

The number of CD3^+^, CD3^+^CD4^+^, and CD3^+^CD8^+^ cells was increased in the vehicle group when compared to the syngeneic group (Figure 3A–C). PEA treatment led to a reduction in the number of these cells when compared to the vehicle group. Regarding the activation, CD3^+^CD8^+^ cells were more activated in the vehicle group compared to syngeneic, but treatment with PEA reduced the MFI of CD28 in the CD3^+^CD8^+^ cells (Figure 3E). Although the number of CD4^+^CD25^+^ cells and the number of regulatory T cells, stained by CD4^+^CD25^+^FoxP3^+^, were lower in the PEA treated group compared to vehicle (Figure 3F,G), the activation of CD4^+^CD25^+^FoxP3^+^ measured by MFI (H) PAE enhance the fluorescence to Foxp3 compared to vehicle treated group. There was no difference between the CD3^+^CD4^+^CD62L^high^, CD3^+^CD4^+^CD62L^low^, CD3^+^CD8^+^CD62L^high^ and CD3^+^CD8^+^CD62L^low^ in the syngeneic compared to the vehicle or the vehicle compared to the PEA treated group (Figure 3I–L).

**FIGURE 4:**
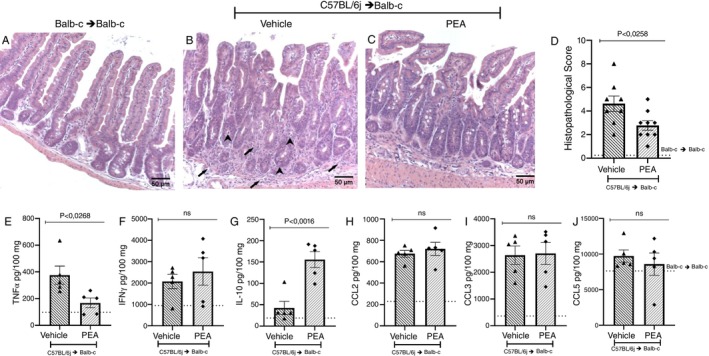
PEA protects the intestine by reducing damage and increasing IL‐10. Slices of intestine of the syngeneic group and allogeneic recipient mice treated with vehicle or PEA (A–C) on the 7th day after disease induction, were fixated and stained with H&E for analysis of their (D) histopathological score. Samples of the intestines were collected, processed and the ELISA assay was performed, and the concentration of the chemokines (E) TNF‐α, (F) IFN‐γ, and (G) IL‐10 and the cytokines (H) CCL2, (I) CCL3 and (J) CCL5 were verified. Treatment was performed from Day 0 until the end of the experiment. Groups were considered significantly different when *p* < 0.05, compared to the allogeneic group. Assessed by unpaired parametric two‐tailed Student's *t* test (*n* = 8–9 for the histopathological score *n* = 5 for the quantification of Inflammatory mediators). The *N* for the histopathological score was result of two experiments.

For histopathological analysis, a blind examination of small intestine slices, stained with H&E, was conducted by a pathologist. The vehicle group presented alteration and loss of crypt architecture and epithelium with moderate edema and hyperemia and increased infiltration of mononuclear cells when compared to the syngeneic transplanted group. Treatment with PEA reduced inflammatory infiltration and preserved crypt architecture, thus protecting the intestine (Figure 4A–D). Additionally, the levels of pro‐inflammatory cytokines and chemokines in the intestine were investigated. The vehicle group had an increase in CCL2, CCL3, TNF‐α and IFN‐γ when compared to the syngeneic group. Treatment with PEA reduced the concentration of TNF‐α and did not alter CCL2, CCL3 or IFN‐γ levels in the intestine. No alteration was observed in the levels of CCL5 (Figure 4E–I). However, the levels of IL‐10 were increased by treatment with PEA (Figure 4J).

### 3.4 Treatment With PEA Reduced Infiltration of CD3 ^+^ CD8 ^+^ Cells, Activation of CD3 ^+^ CD4 ^+^ and CD3 ^+^ CD8 ^+^, TNF‐α Levels, and Damage in the Liver of GVHD Mice

**FIGURE 5:**
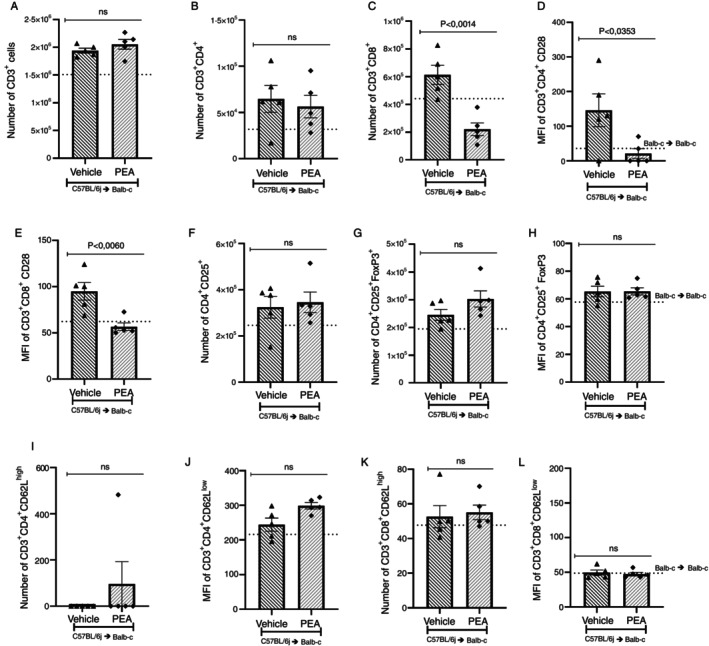
PEA reduced CD3^+^CD8^+^ cells and activation of CD3^+^CD8^+^ and CD3^+^CD4^+^ cells in the liver. The lymphocytic profile was investigated by flow cytometry, determining the number of (A) CD3^+^, (B) CD3^+^CD4^+^ and (C) CD3^+^CD8^+^, and the MFI of CD28 in (D) CD3^+^CD4+ and (E) CD3^+^CD8^+^ lymphocytes. The number of (F) CD4^+^CD25^+^ cells and (G) CD4^+^CD25^+^FoxP3, and MFI of CD4^+^CD25^+^FoxP3. The profile of effector or central memory cells was also investigated in T cells (H) CD3^+^CD4^+^CD62L^high^, (I) CD3^+^CD4^+^CD62L^low^, (J) CD3^+^CD8^+^CD62L^high^ and (K) CD3^+^CD8^+^CD62L^low^. Treatment was performed from Day 0 until the end of the experiment. Groups were considered significantly different when *p* < 0.05, compared to allogeneic group. Assessed by unpaired parametric two‐tailed Student's *t* test (*n* = 5 in all groups).

**FIGURE 6:**
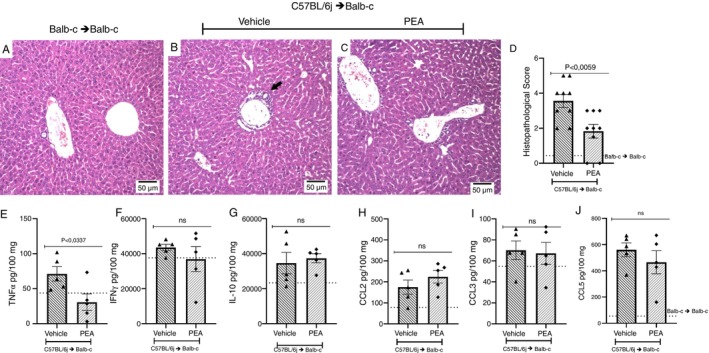
Treatment with PEA reduced damage and TNF‐α levels in the liver of mice subjected to GVHD. H&E‐stained slices of liver from the syngeneic group, and from allogeneic recipient mice treated with vehicle or PEA were examined (A–C). The → indicates inflammatory infiltrate. (D) Histopathological scores of the groups were then compared. The concentrations of cytokines and chemokines were evaluated (E) TNF‐α, (F) IFN‐γ, (G) IL‐10, (H) CCL2, (I) CCL3 and (J) CCL5. Treatment was performed from Day 0 until the end of the experiment. Groups were considered significantly different when *p* < 0.05, compared to the allogeneic group. Assessed by unpaired parametric two‐tailed Student's *t* test (*n* = 9 for the histopathological score *n* = 5 for the quantification of Inflammatory mediators). The *N* for the histopathological score was result of two experiments.

Flow cytometry was also performed to evaluate the inflammatory infiltrate and profile of infiltrated cells into the liver on the 7th day after disease induction. There were no alterations in the number of CD3^+^ and CD3^+^CD4^+^ cells between the vehicle and the treated group (Figure 5A,B). Still, the number of CD3^+^CD8^+^ cells and activation of CD3^+^CD4^+^ and CD3^+^CD8^+^ cells, measured by MFI of CD28, were reduced in mice treated with PEA compared to vehicle (Figure 5C–E). No changes were observed regarding the number of CD4^+^CD25^+^ or FoxP3 cells, neither in the MFI of FoxP3 or in the CD3^+^CD4^+^CD62L^high^, CD3^+^CD4^+^CD62L^low^, CD3^+^CD8^+^CD62L^high^ and CD3^+^CD8^+^CD62L^low^ comparing vehicle and PEA treated group (Figure 5F–L). In liver slices, a pathological analysis found alterations in cellular morphology and diffuse vacuolization with infiltration in periportal and intralobular regions in the vehicle group compared to syngeneic transplanted mice. Treatment with PEA reduced inflammatory infiltrate and damage protecting the liver of GVHD mice (Figure 6A–D) and corroborating the histological findings, the levels of TNF‐α were reduced in the GVHD mice treated with PEA compared to vehicles (Figure 6E). Treatment with PEA did not alter the levels of IFN‐γ and IL‐10, nor did chemokines levels when contrasted with the vehicle group (Figure 6F–J).

### 3.5 The Protective Effect of PEA on Mice Submitted to Experimental Graft‐Versus‐Host Disease Was Dependent on PPAR‐γ Activation

**FIGURE 7:**
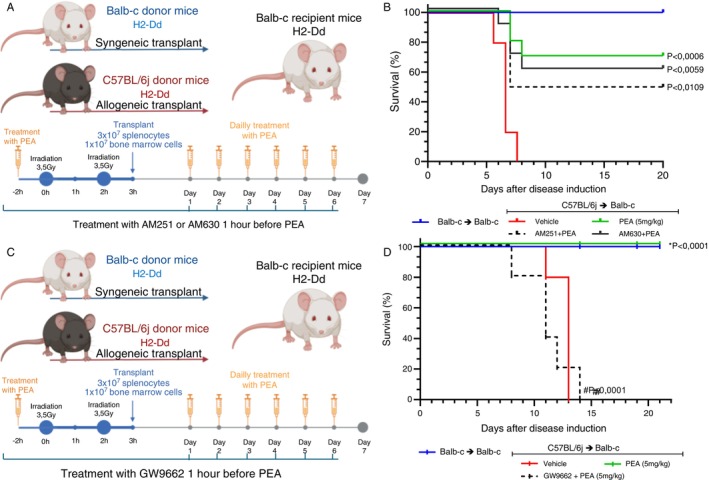
Receptors involved in the effect of PEA treatment on survival of mice submitted to Graft‐versus‐host disease. (A) Procedure of disease induction, previously irradiated BALB‐c mice received either allogeneic or syngeneic splenocytes (3 × 10^7^) and bone marrow cells (1 × 10^7^), thus developing or not GVHD, respectively. GVHD occurs due to differences in MHC I from BALB‐c (H2‐Dd) and C57BL/6J (H2‐Db) mice. GVHD mice received treatment with AM251 or AM630 1 h before PEA treatment. (B) Percentage of survival of mice receiving daily treatment of PEA 5 mg/kg (green line), AM251 1 mg/kg + PEA 5 mg/kg (dashed black line) and AM630 1 mg/kg + PEA (1 mg/kg) (continuous black line) *p < 0.05 compared to vehicle (red line). (C) Procedure of disease induction. GVHD mice received treatment with GW9662 1 h before PEA treatment. (D) Percentage of survival of mice receiving daily treatment of PEA 5 mg/kg (green line), GW9662 1 mg/kg + PEA 5 mg/kg (dashed black line) *p < 0.05 compared to vehicle and ^#^ *p* < 0.05 compared to PEA treatment alone. *n* = 10 results from two different experiments. Treatment was performed from Day 0 until the end of the experiment. Survival was verified by Log‐rank (Mantel‐Cox) test and groups were considered different only when *p* value was < 0,05.

The receptor by which PEA reduces GVHD and increases survival was investigated. The first receptors evaluated were the cannabinoids, CB_1_ and CB_2_. The antagonist of the CB_1_ receptor, AM251, or the inverse agonist of the CB_2_ receptor was administered to mice at the GVHD induction and 1 h before PEA treatment. The treatment with AM251, AM630 and PEA was performed every 24 h until the end of the experiment (Figure 7A). The occupation of the CB_1_ receptor or CB_2_ receptor did not alter the protective effect of PEA on mice submitted to experimental GVHD (Figure 7B). However, the same protocol was repeated to evaluate the PPAR‐γ receptor. The antagonist of PPAR‐γ, GW9662, was administered to mice submitted to experimental GVHD at the GVHD induction until the end of the experiment, once a day, and before PEA treatment (Figure 7C). The occupation of the PPAR‐γ receptor by GW9662 eliminated the protection of PEA treatment on GVHD mice (Figure 7D).

### 3.6 Treatment With PEA Reduced Overall Graft‐Versus‐Host Disease Without Interfering in GVT Response

**FIGURE 8:**
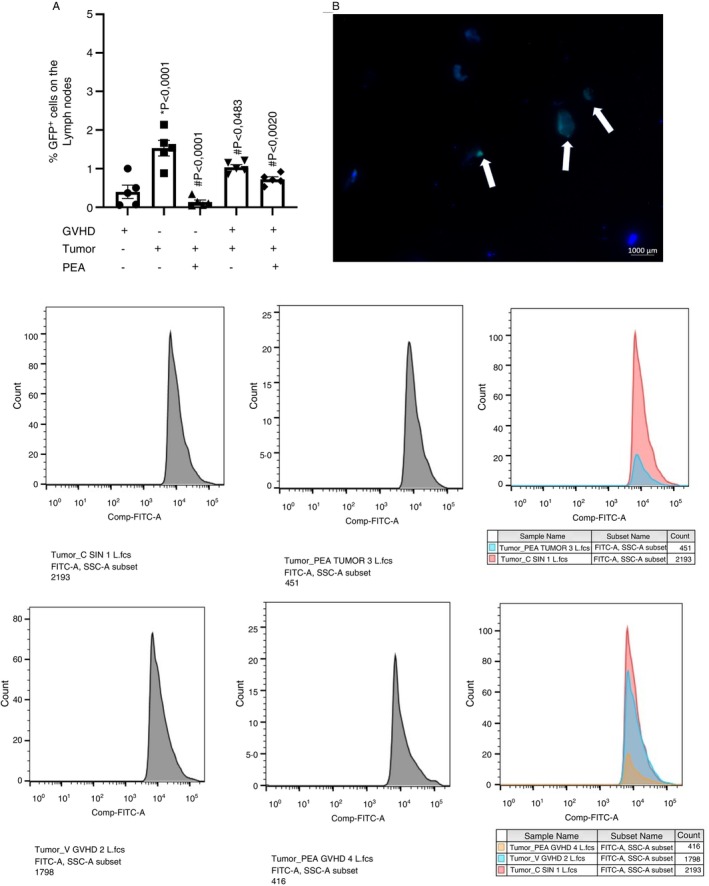
Flow Cytometry of mice lymph nodes, 7 days after disease induction and P815‐GFP+ cells transplant. GVHD was induced by bone marrow cells and splenocytes transplantation. On Day 0 of disease induction 1 × 10^5^ P815‐GFP^+^ cells were injected i.v. Treatment with PEA did not alter the GVT effect. (A) Frequency of P815‐GFP+ cells were represented in dispersion graphs next to flow cytometry shards; *n* = 5 for all groups. *p < 0.05 compared to GVHD group alone. ^#^ *p* < 0.05 compared to tumour group alone. (B) Representative photo of P815‐GFP+ cells on the lymph nodes of mice. The lymph nodes were removed and the cells extracted for cytospin centrifugation and stained with DAPI for the nuclei visualisation. P815‐GFP^+^ cells are pointed by the arrows.

On the same day as disease induction, mice were injected with P815GFP+ tumour cells. After 7 days, their immune response was evaluated using flow cytometry. The control group received only tumour cells and a syngeneic transplant, showing an increase in P815GFP+ cells in the lymph nodes, indicating tumour progression. The vehicle group, which received tumour cells along with allogeneic bone marrow and splenocytes, exhibited a reduction in GFP+ tumour cells compared to the control group. Similarly, PEA treatment did not disrupt the allograft's anti‐tumour response, as tumour presence in the lymph nodes of PEA‐treated mice was significantly reduced compared to the tumour syngeneic group, preserving the GVT effect (Figure 8).

## 4 Discussion

PEA, N‐hexadecanoylethanolamide, is an endocannabinoid lipid mediator belonging to the N‐acylethanolamine phospholipid family [44]. PEA demonstrates significant therapeutic potential for inflammation, primarily due to its ability to modulate the immune system. Therefore, PEA was utilised as a therapeutic strategy in mice subjected to an experimental model of GVHD. In an allogeneic transplantation model, BALB/c mice received spleen and bone marrow cells from C57 mice. Our GVHD model has been widely recognised in the literature as a robust model for drug screening due to its high mortality rate, and protection from death in this model could serve as the first evidence of a promising therapy [45]. Treatment with PEA at doses of 5 or 10 mg/kg reduced the clinical GVHD score from the 12th day after disease induction and almost entirely reduced mortality.

The therapeutic action of PEA has previously been demonstrated in experimental models of Alzheimer's disease, where PEA improved learning and memory due to its anti‐inflammatory and neuroprotective effects [46]. A similar observation was made by D'Agostino et al., where PEA, by activating PPAR receptors, reduced oxidative stress in the central nervous system (CNS), improving learning in mice subjected to an experimental Alzheimer's disease model [47]. PEA has also been tested as a therapeutic strategy for osteoarthritis in rats, where it reduced edema, cartilage degradation, and the production of pro‐inflammatory mediators. The same effect was observed in mice by Impellizzeri et al. [48]. The therapeutic potential of PEA was further demonstrated by Borelli et al., using an experimental colitis model in mice, where PEA reduced intestinal injury and stimulated epithelial regeneration [33].

In human studies, treatment with PEA has been effective in improving the clinical condition of patients with amyotrophic lateral sclerosis, as evidenced by electromyography analysis and pulmonary function assessment [49]. It also showed efficacy in treating acute mania, where PEA combination therapy demonstrated a reduction in YMRS scores, indicating an improvement in manic symptoms and overall clinical status during acute manic episodes [50]. Furthermore, in patients with COVID‐19, PEA reduced systemic inflammation, oxidative stress, and modulated the coagulation cascade, leading to an improvement in the patients' clinical condition [51].

The data presented above corroborate our findings, showing the potential of PEA as an anti‐inflammatory and immunomodulatory tool and the importance of further investigating the mechanisms underlying its actions.

Then, a flow cytometer was used to assess the effect of PEA on the engraftment process. After bone marrow transplantation, the engraftment of the new marrow in the transplant host is necessary. This engraftment is fundamental to the integration and functioning of a new transplanted immune system [52]. Failure in bone marrow or lymphoid organ engraftment leads to transplant failure, which is a pivotal reason for discontinuing trials of a drug considered as a therapy for GVHD [53]. Our results demonstrate that PEA does not interfere with this process as evaluated in the bone marrow and spleen. Our analysis of the lymphocytic profile in the spleen supports our findings on the preservation of the engraftment process. The presence of thriving cells in the spleen after transplantation indicates a functioning immune system, which is essential for preventing opportunistic infections unrelated to the transplant that could negatively impact survival [54].

The spleen is a central lymphoid organ involved in the pathogenesis of GVHD, driving immune cell activation and expansion before their migration to target organs [55]. In this context, it is important to control the activation of effector cells against host antigens without interfering with their activation against tumour cells, thereby maintaining the Graft‐versus‐Leukaemia (GVL) effect. The enhancement of regulatory T cells (Tregs) has been extensively demonstrated as an efficient therapy for GVHD, both in humans and mice, by creating this balance between GVHD and GVL [56, 57, 58]. The expansion of Tregs in the spleen was sufficient to prolong the survival of mice experiencing lethal acute GVHD while maintaining responsiveness to third‐party alloantigens and leukaemia cells [59]. Interestingly, PEA fosters this regulatory environment in the spleen, increasing the number of CD4^+^CD25^+^FoxP3^+^ cells and the mean fluorescence intensity (MFI) for FoxP3^+^ while reducing the activation of CD3^+^CD4^+^ cells, as evidenced by a decrease in MFI for CD28^+^, a co‐stimulatory molecule crucial for antigen presentation and clonal expansion of T cells [60].

Damage to the gastrointestinal tract is a major cause of morbidity and mortality in GVHD, primarily attributable to T cell‐mediated inflammation. Preserving intestinal integrity is crucial for maintaining nutrient absorption and preventing microbiota transmigration [61]. Treatment with PEA effectively reduced tissue damage, inflammatory infiltrates, and preserved intestinal architecture. Regarding the inflammatory infiltrate, there was a reduction in the number of CD3^+^CD4^+^ and CD3^+^CD8^+^ cells, which were found to be less activated, as assessed by CD28 expression. Previous studies from our group have shown that the presence of activated lymphocytes in the intestines is a major contributor to intestinal injury [40, 41, 60, 62]. Additionally, this issue has been extensively discussed in the GVHD literature, and it is now widely accepted that the presence and activation of CD3^+^CD8^+^ cells drive intestinal injury in GVHD [63, 64, 65, 66, 67].

Similarly, CD3^+^CD4^+^ infiltration in the gastrointestinal tract initiates Th1‐mediated proinflammatory cytokine production, exacerbates pathological damage, and increases mortality [68]. In most murine models, CD4^+^ T cells are critical to the induction of GVHD, either by producing IL‐2, which mediates robust allospecific CD8^+^ T‐cell proliferation, or by generating effector proteins such as TNF‐α [69].

For CD4^+^ and CD8^+^ T cells, PEA treatment reduced the upregulation of CD28 expression. CD28 is well characterised as the most effective co‐stimulatory molecule expressed by naïve and activated T cells. Co‐stimulation through CD28 regulates multiple aspects of T cell function, including cytokine secretion, proliferation, and cell survival [70, 71]. Moreover, the CD28 signal, which is responsible for T cell activation [72, 73, 74, 75], can be enhanced by TNF‐α [76, 77].

Using CD28‐deficient mice, it was demonstrated that CD28 co‐stimulation plays a critical role in the development of GVHD [60, 78, 79], and CD28 blockade ameliorated GVHD mediated by both CD4^+^ and CD8^+^ T cells [80, 81]. In CD4^+^ cells, CD28 signalling positively regulated T cell responses to alloantigens and supported GVHD development in an additive or synergistic manner by reinforcing the CD8^+^ effector response. Its blockade resulted in the suppression of GVHD [60]. Additionally, Nurieva et al. showed that in the absence of positive co‐stimulation mediated by CD28, negative co‐stimulatory molecules, including CTLA‐4 and PD‐1, actively instruct T cells to develop into tolerant cells, characterised by inactivation of intrinsic signalling and transcriptional programmes [82].

In addition to reducing the expression of co‐stimulatory molecules critical for T cell activation, treatment with PEA increased the fluorescence intensity of FoxP3 in CD4^+^CD25^+^ cells, thereby promoting immune response regulation, including within the intestine.

The anti‐inflammatory activity of PEA was also demonstrated by Wang et al., 2014 and their research group using a radiation‐induced intestinal inflammation model [83]. The treatment led to improvements at the lesion site, including a reduction in overall radiation‐induced structural damage, intestinal wall thickness, collagen deposition, and neutrophil influx in irradiated intestinal areas.

Similar results were observed in the context of colitis, a type of intestinal inflammation. Borrelli et al. demonstrated the anti‐inflammatory potential of PEA in an experimental mouse model, which resulted in reduced edema and erosion areas, leukocyte infiltration, and intestinal permeability while also stimulating colonic cell regeneration [33]. According to the authors, these effects involved the action of CB_2_, GPR55, and PPARα receptors [33]. Additionally, Peritore et al., using a PEA/Pol‐co compound that combines PEA with polydatin, a fatty acid derivative, to treat murine colitis, observed anti‐inflammatory activity, improved clinical signs, reduced pro‐inflammatory cytokines IL‐1β and TNF‐α, myeloperoxidase (MPO), malondialdehyde (MDA), nitrotyrosine expression, PARP protein, ICAM‐1 and P‐selectin, as well as increased levels of SIRT‐1, heme oxygenase 1 (HO‐1) expression and Nrf2 [84]. This protective effect was not species‐dependent, as evidenced by the anti‐inflammatory effect of PEA‐OXA observed in an experimental zebrafish model of colitis, as reported by Di Paola et al. [85]. The compound was found to reduce intestinal damage, mucus production, and the expression of inflammatory and endoplasmic reticulum stress‐related genes.

TNF‐α is a major inflammatory cytokine associated with damage in GVHD target organs, and its control is crucial for promoting protection in GVHD‐affected mice [86]. PEA treatment reduced TNF‐α levels in the intestines of GVHD mice and promoted an increase in IL‐10.

The effect of PEA on TNF‐α production has been demonstrated in previous studies. Using a spinal cord injury model, Genovese et al., showed that intraperitoneal administration of PEA in mice reduced neutrophil infiltration and TNF‐α levels, improving functional deficits [87]. Similar results were observed by Paterniti et al., who showed that the reduction in neutrophil infiltration and TNF‐α levels was related to the activation of PPAR receptors by PEA [42]. Other studies have shown that PEA treatment can reduce neural injury by controlling TNF‐α levels [29, 88, 89, 90].

The reduction of TNF‐α levels by PEA treatment was also observed by Petrosino et al. using a rat paw edema model and neuropathic and inflammatory pain models, respectively [44, 91]. De Filippis et al. demonstrated similar effects in a model of chronic granulomatous inflammation in rats, and Impellizzeri et al. observed these effects in the treatment of inflammatory arthritis in induced mice [48, 92]. Most of these studies suggested that reducing NF‐κB by PEA is a key step in lowering TNF‐α.

Another vital cytokine to consider in the inflammatory response associated with GVHD is IL‐10. IL‐10 is a regulatory interleukin associated with protective roles and has been linked to a reduction in disease severity in humans [93]. Impellizzieri et al. demonstrated the ability of PEA to increase IL‐10 levels and positively regulate the antioxidant response in a vascular dementia model in mice [94]. Additionally, Wang et al. observed that PEA could inhibit pathways controlling mast cell‐derived cellular immune responses and enhance the anti‐inflammatory signalling of IL‐6 and IL‐10 in the intestines of mice exposed to radiation [83].

The source of IL‐10 in our model remains undetermined. However, we hypothesise that Treg cells (FoxP3^+^) play a crucial role in IL‐10 production, as described by Berg et al. [38, 43]. Although PEA treatment reduced the overall T cell population in the intestine, the intensity of FoxP3 fluorescence, measured by mean fluorescence intensity (MFI), was enhanced. The expression of the transcription factor FoxP3 is essential for the regulatory activity of Tregs. Thus, after treatment with PEA, this population could be responsible for IL‐10 production.

In the liver, a similar protective profile was observed, with reduced CD3^+^CD8^+^ infiltration and decreased activation of both CD3^+^CD8^+^ and CD3^+^CD4^+^ T cells, as assessed by the MFI of CD28. This finding is consistent with the observed reduction in TNF‐α secretion, similar to the effects seen in the intestine. Panoskaltsis‐Mortari et al. found that liver involvement in GVHD typically occurs after intestinal damage, with T cells first migrating to and damaging the intestine before being recruited to the liver [55]. The protective role of PEA in the liver was demonstrated by Hu et al. in a mouse model of non‐alcoholic steatohepatitis (NASH) [95]. Treatment with PEA attenuated the progression of NASH, alleviated oxidative stress, and reduced the expression of genes related to lipid metabolism and inflammatory mediators, such as iNOS, TNF‐α, CCL5, CCL2 and the activation of the NALRP3 inflammasome [95]. The authors attributed the effects of PEA to the activation of PPAR receptors.

Although the anti‐inflammatory and immunomodulatory effects of PEA are extensively described in the literature, studies exploring its mechanisms of action and therapeutic activity are scarce. Therefore, we evaluated which receptor might be involved in the observed protection against mortality in mice subjected to GVHD. Our results indicate that the protective effect of PEA can be entirely abolished by antagonism of the PPAR‐γ receptor, while the same is not observed with antagonism of CB receptors.

In a nociception model induced by formalin injection, Calignano et al. discussed that the effects of PEA, which are dependent on CB_2_ receptors, might result from the potentiation of AEA, which would bind to CB_2_ [96]. In an inflammatory hyperalgesia model induced by carrageenan injection, Jhaveri et al. described this potentiation effect as the ‘entourage effect’, a term that elucidates the synergistic interaction between cannabinoids [97]. Therefore, PEA could enhance both the concentration and effects of endogenous AEA. In a previous study, our group demonstrated that AEA treatment reduced the mortality of mice subjected to the experimental GVHD model, and this effect was associated with CB_2_ receptor activation [43]. Consequently, one of our study's hypotheses was that PEA could increase AEA concentrations, thereby improving the survival of mice experimentally subjected to GVHD. However, when cannabinoid receptors were blocked with their antagonists or inverse agonists, PEA continued to exert its effect on survival, indicating that this effect was not directly associated with CB receptors.

In previous studies, it was observed that Δ9‐THC and CBD, which share the same molecular formula, behave differently in their interactions with cannabinoid receptors [38, 98]. Similarly, despite their high molecular similarity, we observed that AEA and PEA exhibited distinct interactions with cannabinoid receptors in relation to the increased survival of animals with GVHD, with AEA being dependent on CB_2_. Conversely, due to the observed PEA effect being similar to the AEA, the antagonism of the CB_2_ does not interfere with survival.

Moreover, PEA displays a certain degree of pharmacological promiscuity, capable of interacting with various receptors beyond cannabinoid receptors. Some examples include the vanilloid receptor type 1, PPARs, 5HT receptors, GPRs, among others [99, 100, 101]. In this context, PEA has been extensively studied for its interaction with PPAR, a nuclear receptor that plays a crucial role in regulating inflammation and various metabolic processes. Prior treatment of GVHD mice with the PPAR‐γ antagonist, GW9662, completely abolished the protective effect of PEA on survival rates.

The anti‐inflammatory and neuroprotective properties of PEA administration have been demonstrated by Paterniti et al. using a spinal cord injury model in mice [42]. PEA restored physiological levels of TNF‐α and IL‐1β, but the genetic absence of PPAR‐α or the use of PPAR‐δ or PPAR‐γ antagonists reversed the reduction induced by PEA. PEA also attenuated iNOS expression and restored PPAR‐γ and PPAR‐δ levels in the spinal cord [42]. In another study using a mouse model of intracerebral haemorrhage (ICH), intraperitoneal administration of PEA exhibited anti‐inflammatory activity, improved neurological and motor function, and attenuated NF‐κB, IL‐1β, and TNF‐α. This mechanism was correlated with PPAR receptors [102].

The importance of the PPAR‐γ receptor in GVHD was previously demonstrated through the protection of target organs and reduction in the production of inflammatory mediators in mice with GVHD subjected to treatment with the PPAR‐γ agonist, rosiglitazone [103]. The interaction of PEA with the PPAR‐γ receptor producing anti‐inflammatory, analgesic, and immunomodulatory effects has been previously demonstrated in several different experimental models, such as neuropathic pain [104], in tumour cell apoptosis [105], in models of multiple sclerosis, spinal cord trauma [42] and in intestinal inflammation induced by ischemia and reperfusion [106].

The GVT effect appears after allogeneic HSCT and is dependent on allogeneic donor cells that can eliminate residual malignant cells. This anti‐tumour effect is known as GVL or GVT. It is a critical factor in the medical decision to proceed with HSCT as a therapeutic option. It is important to note that, despite the higher likelihood of developing GVHD, allogeneic transplantation provides a stronger GVL effect than other transplantation types [107]. Therefore, an ideal therapy for patients with GVHD would involve the use of drugs that modulate, rather than suppress, the inflammatory response, thus balancing GVL and GVHD [108]. In our experimental model of GVHD, treatment with PEA was able to abrogate the allogeneic response against the target organ but preserves the GVT response observed in the lymph nodes of GVHD mice injected with tumour cells.

The GVL experiment indicated that PEA treatment did not interfere with the response of allogeneic donor lymphocytes to tumour cells injected into mice. This effect is significant when considering potential pharmacological treatment for GVHD. Although PEA reduced tumour cell proliferation in mice that did not receive allogeneic transplantation, its antitumour effect was not the focus of our study. Further experiments should be conducted to confirm this. However, Pagano et al., showed that PEA could inhibit the proliferation, migration, and cell cycle of colon cancer cells studied in vitro and in vivo [109]. Interestingly, PPAR receptors are also involved in the mechanism of action of PEA on the tumour. PEA‐induced cell cycle arrest in the G2/M phase, possibly through cyclin B1/CDK1 upregulation and induced DNA fragmentation. In addition, Camoglio et al., have investigated the effect of PEA treatment on human neuroblastoma SH‐SY5Y cells, resulting in o increased apoptotic cell death marked by the cleavage of caspase 3 and poly‐(ADP ribose) polymerase (PARP) alongside a decrease in survivin and IKBα levels. Treatment of SH‐SY5Y culture with PEA also increased p38 MAP kinase phosphorylation and programmed death‐ligand 1 (PD‐L1) expression in full cell lysate and surface membranes [110]. In this way, PEA could reduce the proliferation of P815 mastocitoma cells in our model.

The balance between the effector and regulatory components of the immune system also influences the severity of GVHD. The absence of regulatory cell populations has been shown to exacerbate GVHD severity [111, 112], indicating that counterregulatory mechanisms are active during GVHD. However, they are often insufficient to prevent or mitigate the disease [113]. PEA treatment established this regulatory environment by increasing the number of regulatory cells in target organs and elevating IL‐10 levels. It also reduced effector T cells and damaged the intestine and liver. However, effector T cells from the donor are crucial for an effective response against tumours. This response should be preserved when promising therapy is proposed. Despite PEA reducing GVHD, it did not alter the GVT response, demonstrating its potential as an alternative therapy for GVHD.

## 5 Conclusion

The present study demonstrated that PEA treatment increased survival and reduced clinical scores in mice subjected to GVHD. PEA treatment did not interfere with engraftment in the spleen and bone marrow. Still, intriguingly, it increased the number of T regulatory cells and the intensity of Foxp3 expression while reducing the expression of the co‐stimulatory molecule CD28 in CD3^+^CD4^+^ cells. In the intestine, PEA treatment reduced the infiltration of CD3^+^CD4^+^ and CD3^+^CD8^+^ cells, and the CD3^+^CD8^+^ cells also exhibited reduced CD28 expression intensity. Although the number of T regulatory cells decreased, the intensity of Foxp3 fluorescence increased in the intestines of mice treated with PEA. Accordingly, the levels of TNF‐α were reduced, and the IL‐10 levels were increased in the intestines of mice treated with PEA. In the liver, PEA treatment reduced the infiltration of CD3^+^CD8^+^ cells and the co‐stimulatory CD28 expression in CD3^+^CD4^+^ and CD3^+^CD8^+^ cells, as well as TNF‐α levels and tissue damage. The effect of PEA on the survival of GVHD mice was dependent on PPAR‐γ activation but did not rely on CB receptor activation.

## Author Contributions

B.B.B., A.F.S.L., Z.D.T.C., and G.M.F.R., performed experiment. B.B.B., B.M.R., and M.G.M.C, analysed results and drew figures. S.B.A.C., T.R.L.R., V.P., and M.M.T. contributed to the experimental design, with drugs and reagents and analytic tools. B.B.B. and M.G.M.C., designed the research and wrote the paper.

## Conflicts of Interest

The authors declare no conflicts of interest.

## Supporting information


**Figure S1.** Gating strategy. (A) Initial sample separated by granularity and size. (B) Separation of single cells within the granulocyte population. (C) Separation of single cells within the monocyte population. (D) Separation of single cells within the lymphocyte population. (E) Identification of the CD3^+^ cell population. (F) Identification of the CD4^+^ and CD8^+^ cell populations.

**Figure S2.** FoxP3 analysis. (A) Initial sample of intestinal cells with total lymphocyte selection. (B) Identification of CD4^+^ and CD25^+^ cell populations. (C—D) Mean fluorescence intensity of FoxP3 cells within the CD4^+^ CD25^+^ cell population.

**Figure S3.** Flow cytometry analysis of bone marrow engraftment. Representative histogram of H2‐Db and H2‐Dd expression in bone marrow cells. Seven days after transplant, bone marrow was proceeded to flow cytometry analysis. Frequency of H2‐Db and H2‐Dd was accessed by specific surface markers.

**Figure S4.** Flow cytometry analysis of spleen, seven days after disease induction. Analysis of cell surface markers, such as CD3, CD4, CD8 and the activation marker CD28 and CD62l. CD4, CD25 and FoxP3 to characterise T regulatory lymphocytes.

**Figure S5.** Flow cytometry analysis of intestine, seven days after disease induction. Analysis of cell surface markers, such as CD3, CD4, CD8 and the activation marker CD28 and CD62l. CD4, CD25 and FoxP3 to characterise T regulatory lymphocytes.

**Figure S6.** Flow cytometry analysis of liver, seven days after disease induction. Analysis of cell surface markers, such as CD3, CD4, CD8 and the activation marker CD28 and CD62l. CD4, CD25 and FoxP3 to characterise T regulatory lymphocytes.

## Data Availability

The data that support the findings of this study are available from the corresponding author upon reasonable request.
