# Supplementary figures and images for: Palmitoylethanolamide (PEA) Induces an Increase in Spleen Regulatory T Cells, Reduces CD8 + Cells and TNF‐α Levels in Target Organs, and Protects Mice From Graft‐Versus‐Host Disease‐Related Mortality Through PPAR Activation Without Compromising the Graft‐Versus‐Tumour Response
Source: Immunology. 2025 Jun 25;176(3):385–402. doi: 10.1111/imm.70010 (PMC12500389; doi:10.1111/imm.70010)

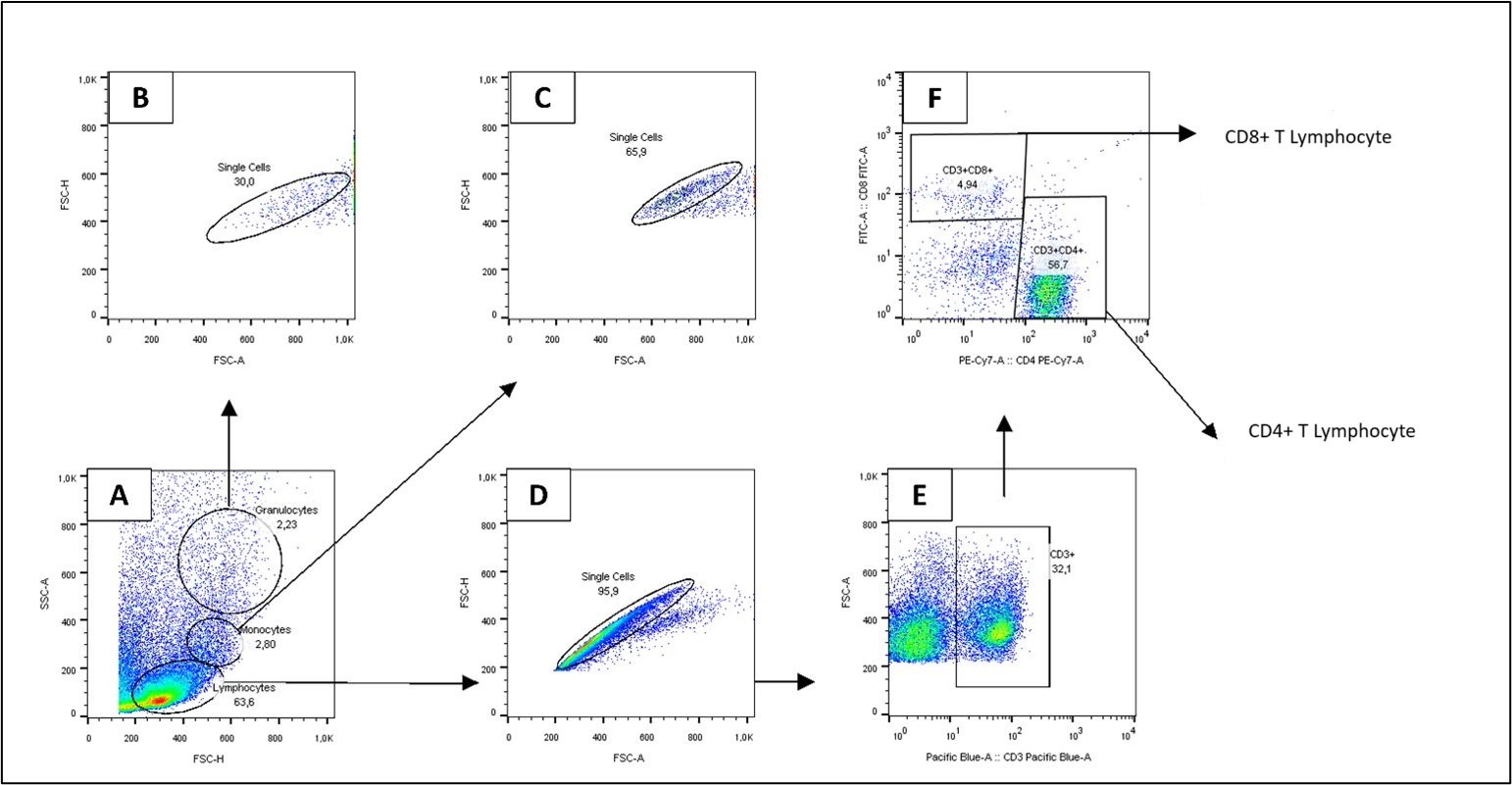

Supplement: Supplementary file 1 — Figure S1. Gating strategy. (A) Initial sample separated by granularity and size. (B) Separation of single cells within the granulocyte population. (C) Separation of single cells within the monocyte population. (D) Separation of single cells within the lymphocyte population. (E) Identification of the CD3+ cell population. (F) Identification of the CD4+ and CD8+ cell populations. [file IMM-176-385-s005.jpg]

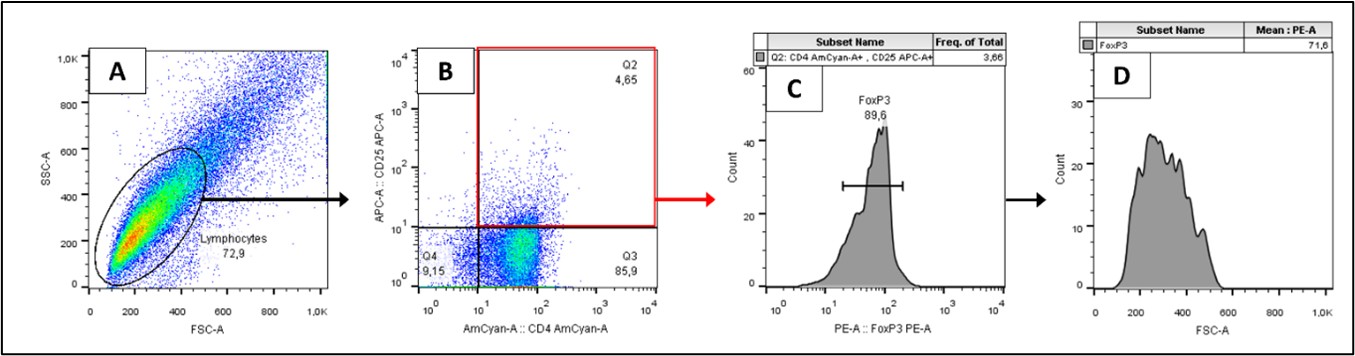

Supplement: Supplementary file 2 — Figure S2. FoxP3 analysis. (A) Initial sample of intestinal cells with total lymphocyte selection. (B) Identification of CD4+ and CD25+ cell populations. (C—D) Mean fluorescence intensity of FoxP3 cells within the CD4+ CD25+ cell population. [file IMM-176-385-s004.jpg]

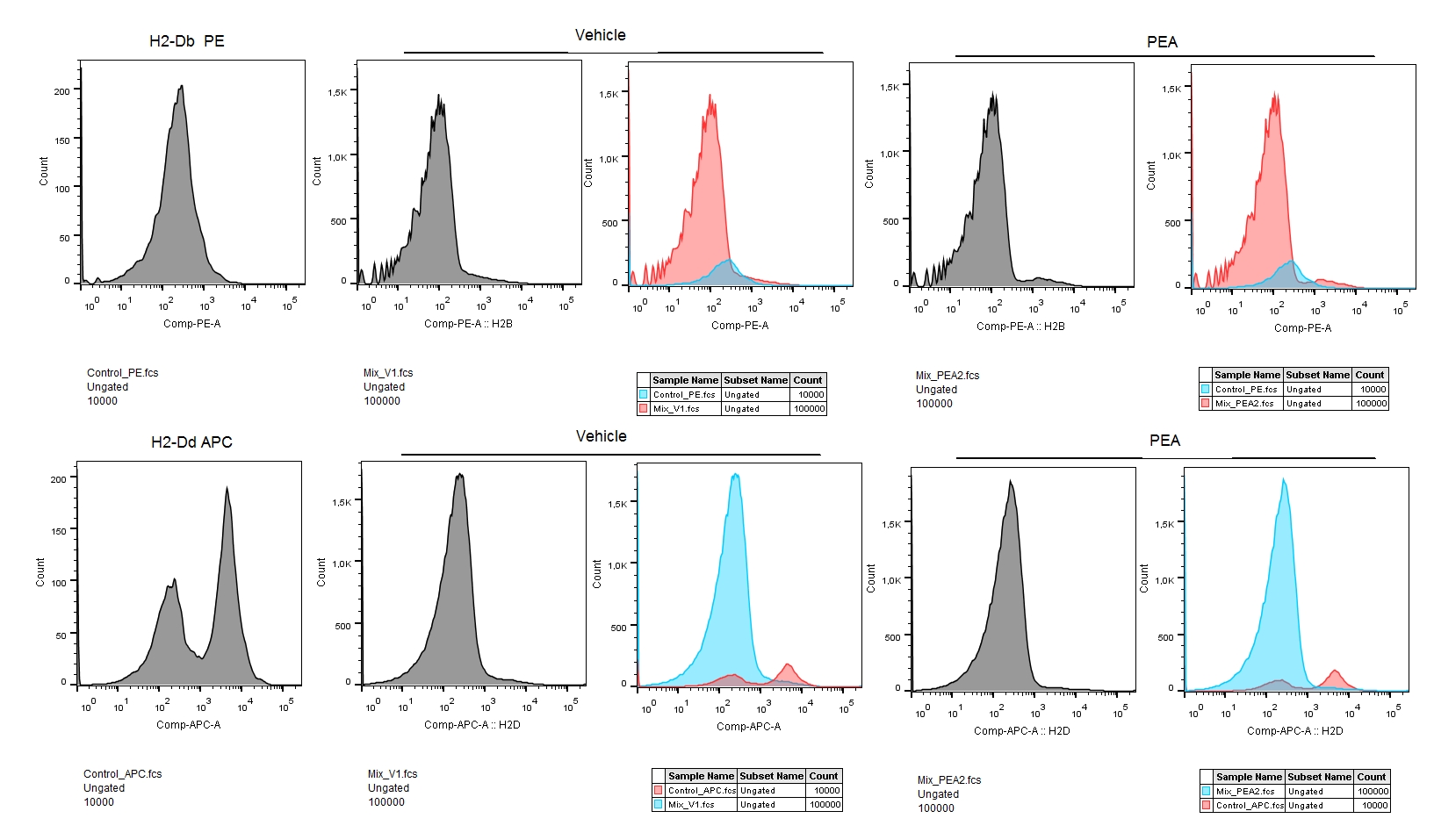

Supplement: Supplementary file 3 — Figure S3. Flow cytometry analysis of bone marrow engraftment. Representative histogram of H2‐Db and H2‐Dd expression in bone marrow cells. Seven days after transplant, bone marrow was proceeded to flow cytometry analysis. Frequency of H2‐Db and H2‐Dd was accessed by specific surface markers. [file IMM-176-385-s006.jpg]

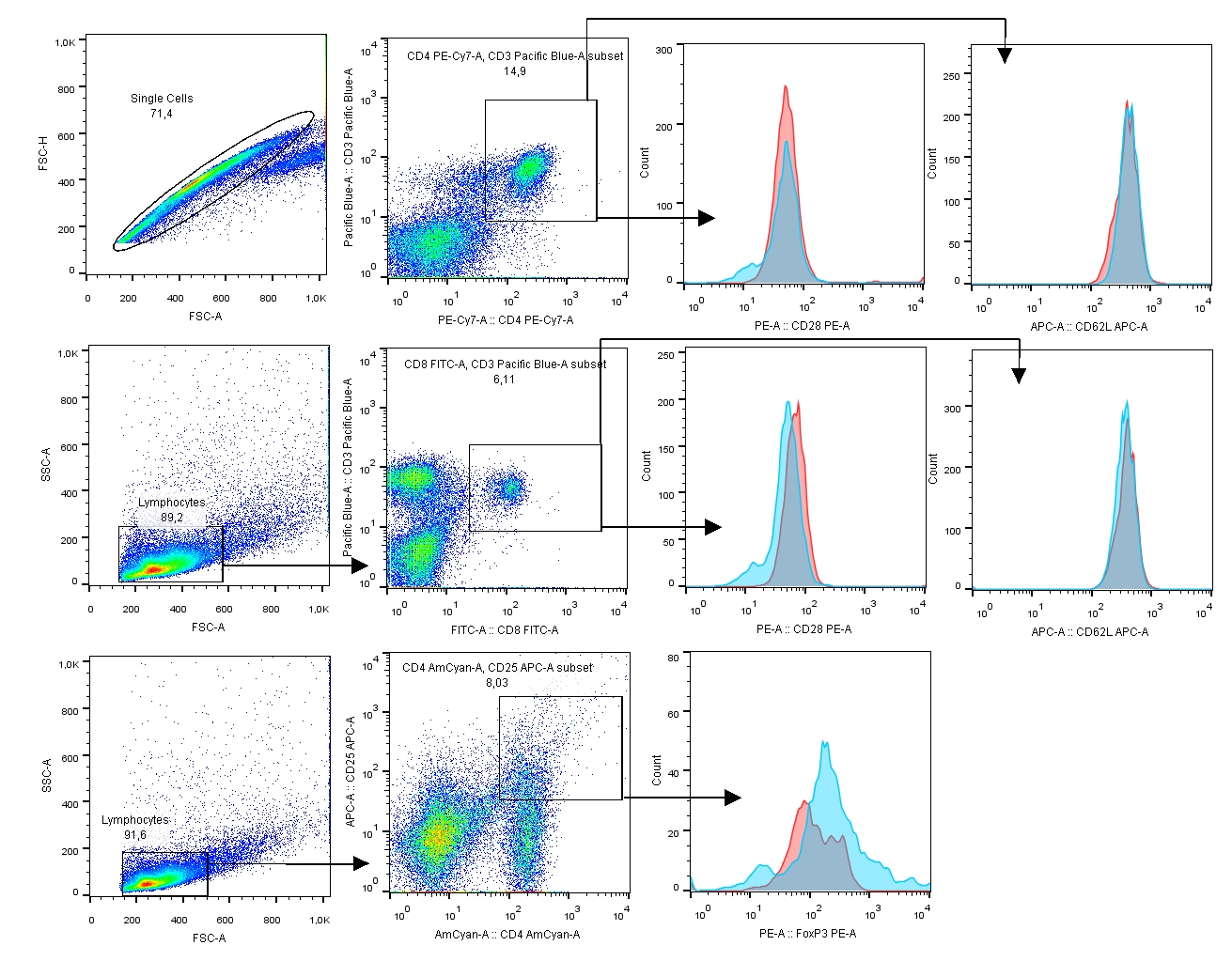

Supplement: Supplementary file 4 — Figure S4. Flow cytometry analysis of spleen, seven days after disease induction. Analysis of cell surface markers, such as CD3, CD4, CD8 and the activation marker CD28 and CD62l. CD4, CD25 and FoxP3 to characterise T regulatory lymphocytes. [file IMM-176-385-s003.jpg]

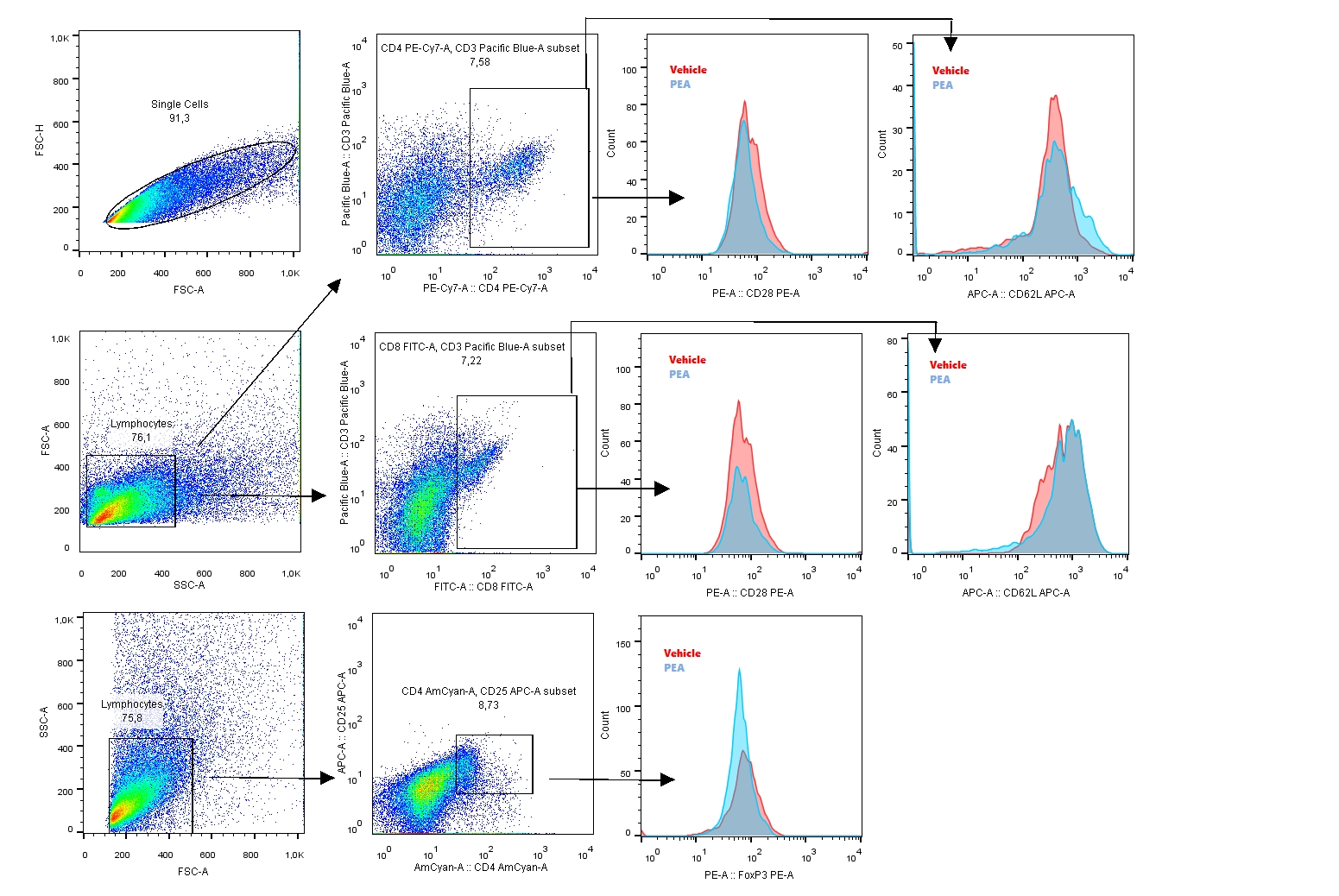

Supplement: Supplementary file 5 — Figure S5. Flow cytometry analysis of intestine, seven days after disease induction. Analysis of cell surface markers, such as CD3, CD4, CD8 and the activation marker CD28 and CD62l. CD4, CD25 and FoxP3 to characterise T regulatory lymphocytes. [file IMM-176-385-s001.jpg]

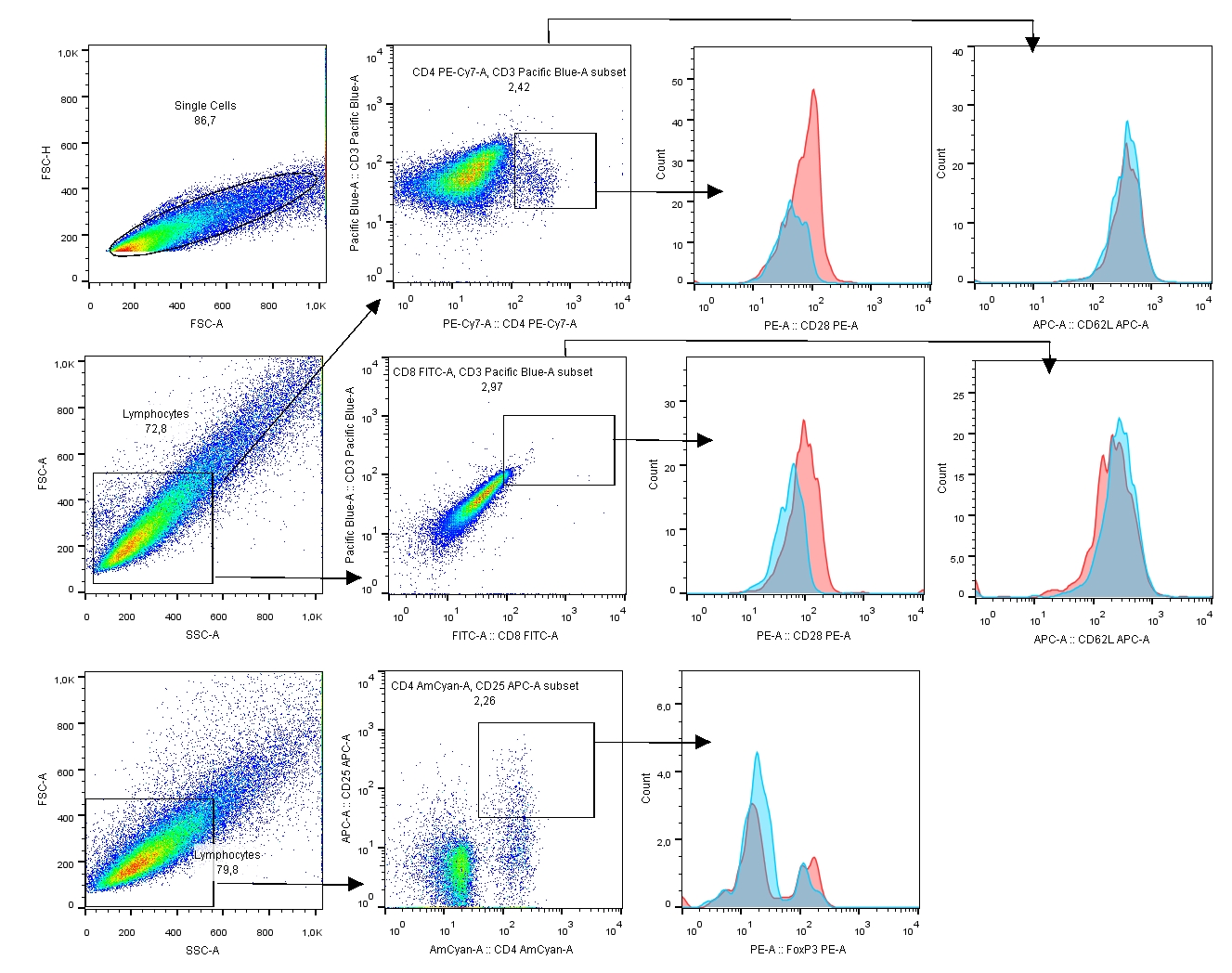

Supplement: Supplementary file 6 — Figure S6. Flow cytometry analysis of liver, seven days after disease induction. Analysis of cell surface markers, such as CD3, CD4, CD8 and the activation marker CD28 and CD62l. CD4, CD25 and FoxP3 to characterise T regulatory lymphocytes. [file IMM-176-385-s002.jpg]
